# Acute-on-Chronic Subdural Hemorrhage Due to Late Vitamin K Deficiency

**DOI:** 10.7759/cureus.34297

**Published:** 2023-01-28

**Authors:** Niraj K Choudhary, Jeevesh Mallik, Kumar Diwakar

**Affiliations:** 1 Department of Neurosurgery, Tata Main Hospital, Jamshedpur, IND; 2 Department of Pediatrics, Tata Main Hospital, Jamshedpur, IND

**Keywords:** craniotomy, breastfed, intracranial bleed, newborn, bleeding disorder

## Abstract

Vitamin K deficiency bleeding (VKDB) is closely associated with the hemorrhagic disease of the newborn (HDN) and can have a late onset, after one week of birth up to six months of age. It is a major concern in developing countries where vitamin K prophylaxis is not often given to newborns and can lead to significant mortality and morbidity. We report a case of a three-month-old child who was exclusively breastfed. He presented with repeated vomiting and was eventually diagnosed as a case of acute-on-chronic subdural hemorrhage. Timely diagnosis and surgical intervention played a key role in ensuring a favorable outcome for the child.

## Introduction

The term hemorrhagic disease of the newborn (HDN) has been replaced with vitamin K deficiency bleeding (VKDB) as it has been observed that bleeding in neonates could be due to causes other than vitamin K deficiency [1]. It is a rare bleeding disorder caused by low levels of vitamin K at birth and affects newborns and young infants [2]. Intracranial hemorrhage is one of its most common presentations, leading to brain damage and even death [3]. Vitamin K, a fat-soluble vitamin, plays an important role in coagulation and is necessary for the production of many clotting factors like II, VII, IX, and X [4]. It has been noted that vitamin K levels transferred through the placenta to a newborn are very low and below the detection levels of 0.02 ng/ml [5]. Also, the gut flora that produces vitamin K for adults is usually not developed in infants [2]. Hence, breastfed babies are more at risk of developing vitamin K deficiency than bottle-fed infants [1].

VKDB can be classified into different types according to the etiology and age of onset. In cases of primary VKDB, no cause other than breastfeeding can be found, while in the secondary type, factors that reduce the vitamin K effect are present, such as malabsorption secondary to hepatobiliary and intestinal diseases, poor intake of vitamin K, or antagonism of vitamin K by drugs. Depending on the age of presentation, VKDB can be classified into three types: early (0-24 hours), classical (one to seven days), and late (one week to six months) syndromes. Any child who has bleeding after seven days of life can fall into the category of late VKDB. These children exhibit no thrombocytopenia, have normal peripheral blood smear, prolonged prothrombin time (PT), prolonged activated partial thromboplastin clotting time (aPTT), and international normalized ratio (INR) >1.8. Rapid correction of PT or cessation of bleeding after vitamin K administration are also characteristics of late VKDB [6].

These patients usually present with seizures, feeding intolerance, and irritability. The majority of cases of late VKDB manifest with intracranial and gastrointestinal bleeding along with skin symptoms that are usually severe [7]. Early surgical management prevents morbidity and mortality, as was evident in our case. We highlight the importance of vitamin K prophylaxis at birth in this case report, as it was lacking in our patient and could have led to an adverse outcome if not treated in a timely manner.

## Case presentation

A three-month-old male child presented to our emergency with a history of repeated vomiting and decreased activity for one day. He had a history of loose stools since the previous day. There was no history of convulsion, head trauma, fever, cold, or cough. The mother gave no history of taking any medications during her pregnancy. The other sibling did not report any such diseases during her childhood. The child had been born by cesarean section at term. He was exclusively breastfed and had normal developmental milestones. The child was not on any medications and there was no history of vitamin K administration after birth.

**Figure 1:**
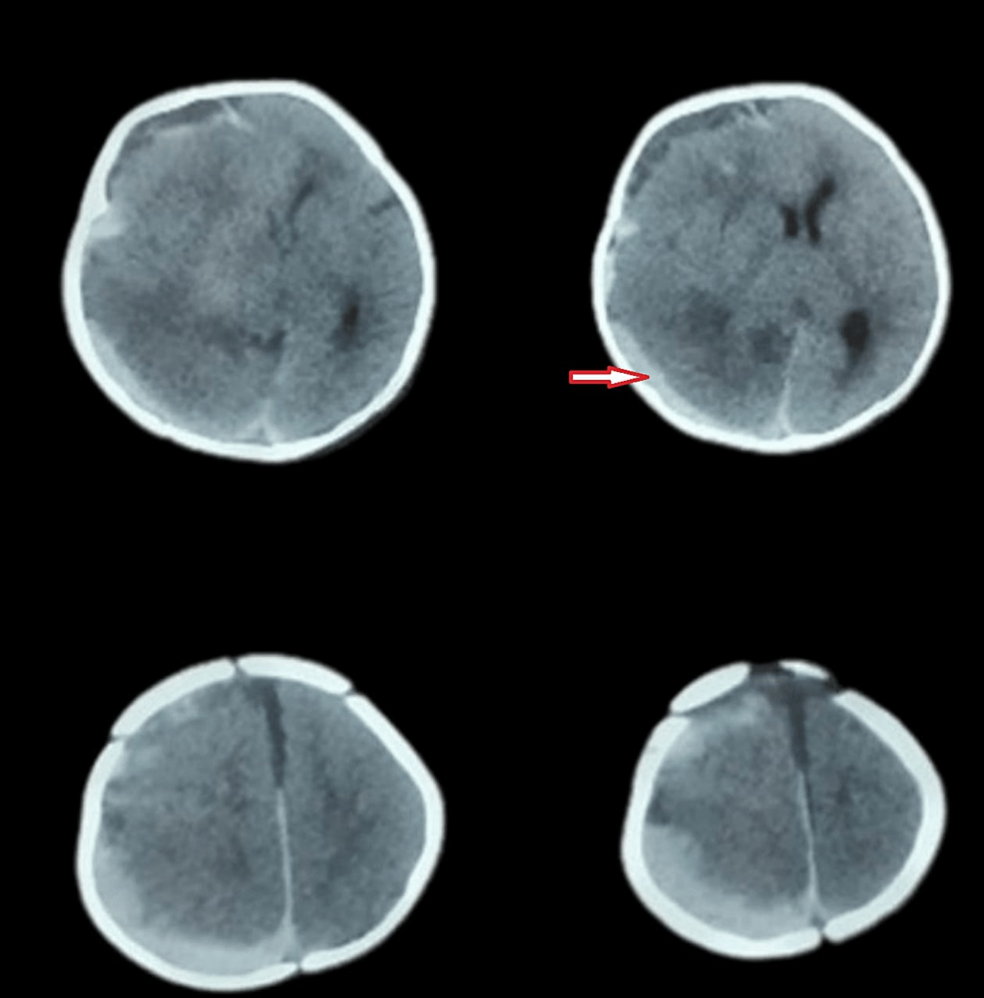
Radiological imaging on admission Orange arrow: CT brain showing subdural hematoma with mass effect CT: computed tomography

On examination, the child was drowsy with a pediatric Glasgow Coma Scale (GCS) score of E3V4M6. The pupils were of normal size and normal in reaction. The child was moving all limbs and no limb weakness was detected. The skin was normal and there were no signs of any bleeding disorder. However, significant pallor was noted. Other systemic examinations were normal. The child was further evaluated for anemia. On investigation, his hemoglobin level was 6.8 g/dl. The platelet count was normal, but the liver function tests were deranged. The total bilirubin was 3.75 mg/dl, aspartate transaminase (AST) was 48.7 IU/liter, and alkaline phosphatase (ALP) was 723 IU/liter. The prothrombin time was also elevated, at more than 90 seconds. Vitamin K in the dose of 5 mg intravenously was administered preoperatively and the PTT decreased to 28 seconds. Packed red blood cells and fresh frozen plasma transfusions were given. After 24 hours of hospital stay, the child's condition deteriorated and he developed anisocoria with right ptosis and tense bulging anterior fontanelle. At this stage, urgent radiological imaging was advised. CT brain showed right-sided acute-on-chronic subdural hemorrhage with mass effect and midline shift (Figure 1).

The child was immediately taken up for emergency burr hole craniotomy with evacuation of subdural hemorrhage. Postoperatively, the child was ventilated and gradually weaned off when his condition improved. During the postoperative period, blood and fresh frozen plasma transfusions were given. Vitamin K was administered intravenously daily for five days and, in due course, the coagulation parameters become normal (Table 1).

**Table 1:** Blood investigations during the course of treatment

Blood investigation	Admission day 1	Postop day 2	Day 3	Day 4	Day 14
Hemoglobin (g/dl)	6.8	6.6	8.8	13.3	12
Platelet count (lac)	2.41	2.93	2.98	2.15	2.5
Total bilirubin (mg/dl)	3.75	-	-	10.18	0.9
Prothrombin time (sec)	>90	11.8	10.9	11.4	15

**Figure 2:**
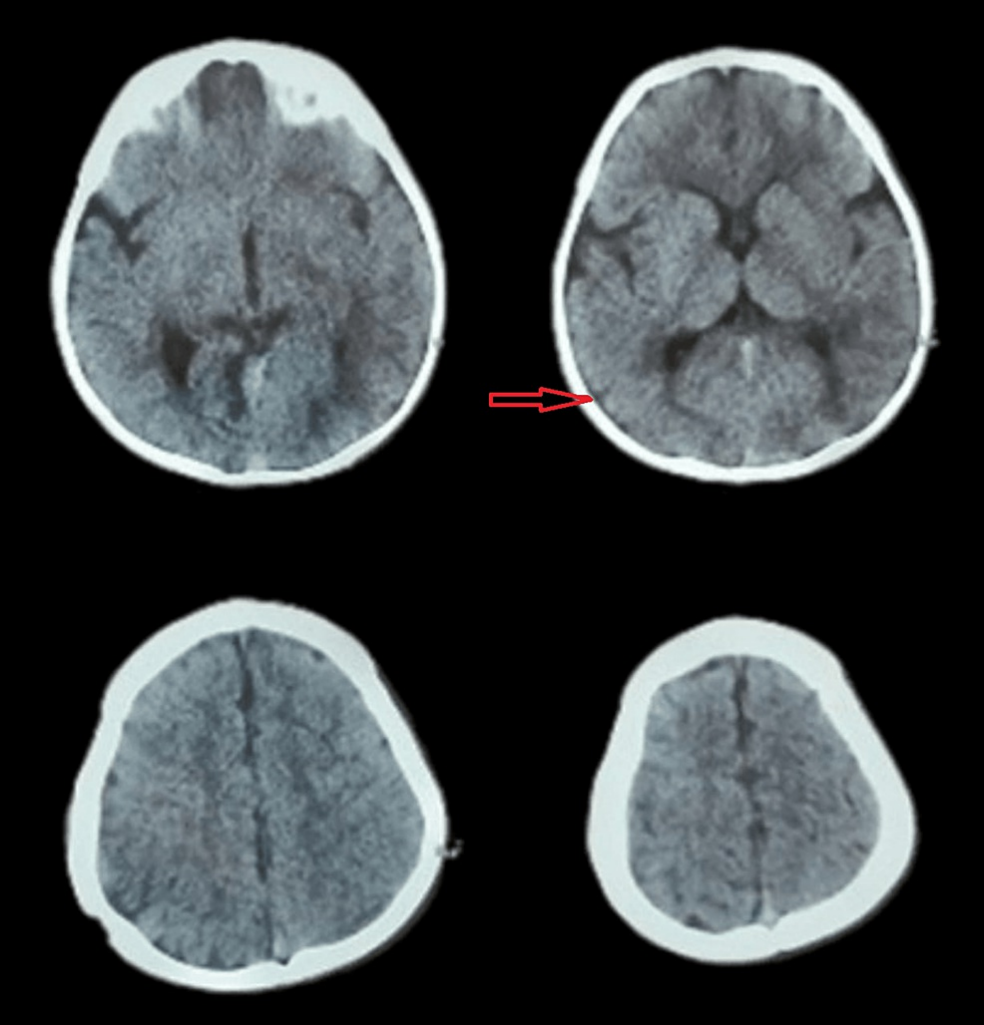
Radiological imaging post-surgery Orange arrow: CT brain showing the resolution of subdural hematoma CT: computed tomography

At the follow-up after one month, a repeat CT brain was done, which showed the resolution of subdural hematoma, confirming a good outcome (Figure 2).

## Discussion

It is a well-known fact that human breast milk has a low concentration of vitamin K, which may increase the incidence of VKDB disorders. It is more prevalent in developing countries where exclusive breastfeeding is often advocated and practiced. Late VKDB is usually seen during the second week of life and is an important cause of morbidity and mortality in infants due to intracranial hemorrhage.

As per several studies in the literature, the incidence of late VKDB ranges from 10.5 to 80 per 100,000 live births when vitamin K prophylaxis is not given [4], and this decreased to the range of 0.24 to 3.2 cases per 100,000 live births when intramuscular vitamin K prophylaxis was given [7]. European and Asian countries have reported an occurrence rate varying from 4.4 to 7.2 cases per 100,000 births. In contrast, studies from the eastern part of the world report a higher incidence ranging from 25 to 80 cases per 100,000 births. Late-onset VKDB is a preventable disorder, and the administration of intramuscular injections of vitamin K at birth should be advocated. The cases have decreased significantly in developed countries due to the extensive use of intramuscular vitamin K prophylaxis at birth. According to the literature, the incidence has decreased from seven to 1.1 per 100,000 in the Netherlands [8]. Another published study from Turkey reported neurological findings in 73% of cases of VKDB and mortality as high as 33% [9]. In contrast, a study from India reported that 71% of cases of VKDB had intracranial hemorrhage as the most common symptom and the mortality rate was about 5% [10].

A prolonged PT and aPTT, which are corrected after vitamin K administration, are the keys to the diagnosis of VKDB. Treatment of these bleeding disorders includes vitamin K administration, which normalizes PT rapidly. In severe cases of bleeding, the administration of fresh frozen plasma and prothrombin concentrate is recommended.

In our case, we detected acute-on-chronic subdural hemorrhage on the CT brain, which is an uncommon presentation of VKDB. This was evident from the review of literature as very few cases of chronic or subacute subdural hematoma in VKDB have been reported to date. Most of the reported cases involved intracerebral bleeding or acute subdural hemorrhage. The child in our case had already developed right-sided ptosis and anisocoria signifying transtentorial herniation leading to third cranial nerve involvement. The child was critical, and prompt surgical intervention after a timely diagnosis saved his life. Another challenge was operating on a child with an altered coagulation profile. Proper therapeutic management along with blood and fresh frozen plasma transfusions helped in normalizing blood values. In this case, the cause seems to be vitamin K deficiency due to exclusive breastfeeding, which got aggravated due to diarrhea.

## Conclusions

The administration of vitamin K post-delivery is routinely advocated and practiced. Its deficiency may lead to VKDB presenting as significant intracranial hemorrhage, which could lead to significant morbidity and mortality. High clinical suspicion and basic investigations can help in the diagnosis of VKDB. Proper medical management with a timely surgical intervention leads to good outcomes with no neurological deficits.
